# Counteracting forces of introgressive hybridization and interspecific competition shape the morphological traits of cryptic Iberian *Eptesicus* bats

**DOI:** 10.1038/s41598-022-15412-2

**Published:** 2022-07-08

**Authors:** Pedro Horta, Helena Raposeira, Adrián Baños, Carlos Ibáñez, Orly Razgour, Hugo Rebelo, Javier Juste

**Affiliations:** 1 CIBIO, Centro de Investigação em Biodiversidade e Recursos Genéticos, InBIO Laboratório Associado, Universidade do Porto, Campus de Vairão, 4485-661 Vairão, Portugal; 2 Departamento de Biologia, Faculdade de Ciências, Universidade do Porto, 4099-002 Porto, Portugal; 3 OII – Observatório Inovação Investigação, Seia, Portugal; 4 University Pablo de Olavide, Sevilla, Spain; 5 Departmento de Ecología Evolutiva, Estación Biológica de Doñana (CSIC), Avda. Américo Vespucio 26, 41092 Sevilla, Spain; 6 CIBER de Epidemiología y Salud Pública, CIBERESP, Madrid, Spain; 7 Biosciences, University of Exeter, Exeter, UK; 8 ESS, Polytechnic Institute of Setúbal, Setúbal, Portugal Campus do IPS - Estefanilha, 2910-761 Setúbal; 9 BIOPOLIS Program in Genomics, Biodiversity and Land Planning, CIBIO, Campus de Vairão, 4485-661 Vairão, Portugal

**Keywords:** Speciation, Zoology, Evolutionary ecology

## Abstract

Cryptic species that coexist in sympatry are likely to simultaneously experience strong competition and hybridization. The first phenomenon would lead to character displacement, whereas the second can potentially promote morphological similarity through adaptive introgression. The main goal of this work was to investigate the effect of introgressive hybridization on the morphology of cryptic Iberian *Eptesicus* bats when facing counteracting evolutionary forces from interspecific competition. We found substantial overlap both in dentition and in wing morphology traits, though mainly in individuals in sympatry. The presence of hybrids contributes to a fifth of this overlap, with hybrids showing traits with intermediate morphometry. Thus, introgressive hybridization may contribute to species adaptation to trophic and ecological space responding directly to the macro-habitats characteristics of the sympatric zone and to local prey availability. On the other hand, fur shade tended to be browner and brighter in hybrids than parental species. Colour differences could result from partitioning of resources as an adaptation to environmental factors such as roost and microhabitats. We argue that a balance between adaptive introgression and niche partitioning shapes species interactions with the environment through affecting morphological traits under selection.

## Introduction

The evolutionary history of species under preliminary stages of macroevolution moves through complex forces, leading to incongruities in phylogenetic inferences and species classification^1^. Divergence is frequently discussed as involving mainly stages of allopatry^2,3^. However, historical environmental fluctuations^4^ mediate iterations between periods of allopatry and sympatry^5^. When past allopatry was not sufficient to eliminate or significantly reduce niche overlap, closely-related species may coexist stably during secondary contacts under antagonistic evolutionary forces, imposed for example by hybridization and niche partitioning^6^. On one hand, under sympatry, genetic material can be transferred through introgressive hybridization with potential evolutionary convergence^7^. On the other hand, ecological interactions, such as competition, predation or parasitism may produce exclusion or promote divergence as a response^8^.

When hybridization between lineages presents adaptive advantages about the parental lines resulting in increased fitness, the genetic pools will face a process of adaptive introgression. This has been described as the main evolutionary response to natural hybridization^9,10^. Introgressed alleles potentially associated with well-adapted traits^11–13^ are introduced into the gene pool of the recipient species by backcrossing and fixed by natural selection^7^. Therefore, introgressed species can jump directly to new adaptive optimum, bypassing intermediate steps^11,14^. By allowing closely-related species to share particularly beneficial traits, adaptive introgression is sometimes understood as a homogenizing process promoting evolutionary convergence^15,16^.

Recent studies have shown important phenotypic expression of adaptive introgression in morphological traits of many taxa. Introgressed morphological traits were reported to promote dehydration/water-loss resistance to warmer or colder climates in plants, for example by increasing the number or size of leaves, branches or roots under desert conditions^9^. Moreover, changes in body width, head shape or eye size were shown to promote higher fitness in some fishes^17^, while larger heads with stronger bites and larger testes promoted better sexual performance in reptiles^18^. The introgression of morpho-physiological traits also promotes higher fitness in some rodents in environments with lower precipitation and temperatures^19^. Ancient events of adaptive introgression have even allowed humans to adapt to island environments by decreasing their size^20^. Colour change through adaptive introgression was reported in insects^13,14^, mammals^21^ and fishes^22^. Colour change is frequently associated with increasing mating performance^18,23^, better mimicry capacity^24,25^, higher success in escaping predators^26^ and in exploring new habitats^22^ and as responses, for example, to climate change^26,27^.

As an opposite evolutionary force, competition may lead species or lineages to avoid each other and to occupy a narrower and differentiated optimum and set of conditions. If this effect is durable and prevalent enough, then it may lead to a species-specific divergent trait displacement due to differential exploitation of resources^28,29^.

Competition has been reported to drive divergence in phenological traits, like changing floral shape of angiosperms in response to altitudinal gradients^30^, and to promote functional and biomechanical differences in jaw closure of salamanders^31^. Moreover, competition has been shown to play a role in beak size divergence and larynx morphology in birds, both relevant traits for diet adaptations and song characteristics, thus influencing sexual selection and species recognition^32^. Head morphology and size divergence were also described as being the result of competition in spadefoot toads, in response to omnivore or carnivore diet^33^.

Despite the relevance of morphological traits for species adaptive capacity and individual fitness, the impact of hybridization on morphological traits when occurring in sympatry or parapatry remains largely unknown. Cryptic species are an extreme case among closely-related taxa for being morphologically identical but genetically distinct^34^. Due to their phylogenetic proximity and partial niche overlap, as well as morphological similarity and a high potential for hybridization, they represent excellent case studies for the effect of antagonistic evolutionary processes on morphological trait displacement, particularly when associated with sympatric or parapatric distributions^35,36^.

Notably, in the Iberian Peninsula, over 20% of known bats species show cryptic diversity^37,38^. Some of the cryptic species pairs exhibit high genetic divergence, such as *Eptesicus serotinus*^39^ and *E. isabellinus*^40^, which show over 16% divergence in the mtDNA Cytochrome b gene^37^. A comparative recent study supports a more pronounced and geographically structured intraspecific genetic variation within *E. isabellinus*^41^. This species has experienced a rapid post-glacial expansion earlier, heading northwards until occupying its current range. *Eptesicus serotinus* is thought to have expanded southwards from Central Europe, likely arriving later than *E. isabellinus* to the ecotone of central Iberia where both species meet^41^. Currently, *E. serotinus* is distributed along the Atlantic region of Iberia (colder and wetter) while *E. isabellinus* mainly occurs in the Mediterranean region (hotter and drier)^42^. Across their ranges, the two species geographically avoid each other, indicating that interspecific competition likely shaped their broad-scale distributions^43^. With a vast allopatric distribution, the species share a restricted contact zone, where they interbreed^41^. Although nuclear markers identified a male‐mediated hybridization, there is no evidence of mitochondrial introgression, contrary to what is known for other *Eptesicus*^44^. Moreover, ongoing hybridization is asymmetric, occurring mainly from *E. isabellinus* to *E. serotinus* (28% hybrids in *E. serotinus* colonies and 2.7% in *E. isabellinus* colonies)^41^.

This study aims to understand the effect of this introgressive hybridization on morphological traits of the two cryptic Iberian *Eptesicus* bats in face of counteracting evolutionary forces from the interspecific competition. We addressed three main questions: (1) Are there significant morphological differences between cryptic *Eptesicus* species?; (2) Could their overlap of morphological traits (wing, dentition and colour) be greater in sympatry due to introgressive hybridization?; (3) What is the contribution of the hybrids to the morphological overlap in sympatry assessed through classification statistics? Clarifying these objectives could elucidate how potential natural gene transfer through introgression can act as a counteracting force for interspecific competition by shaping the morphological traits of parental species in sympatry.

## Methods

### Study area

We searched for *Eptesicus* bat colonies between 1998 and 2014 along a North–South gradient across the Iberian Peninsula, including allopatric areas of only *E. serotinus* in the north, a central region of the sympatric zone and allopatric areas of *E. isabellinus* in the south. Field sampling methods were described in previous publications^41,42^. Bats included in our sample were caught in breeding colonies (females) before births. Thus, all caught individuals can be considered adults because they were at least one year old and had already achieved their sexual maturity. The location of all sampled colonies was also described in previous publications^41^ (see Supplementary Fig. S1). The methods were performed following relevant guidelines and regulations and approved by the Ethical committee at the EBD–CSIC (Estación Biologica de Doñana, Consejo Superior de Investigaciones Científicas) that granted protocol approval. The study was carried out in compliance with the ARRIVE guidelines.

### Sampling design

Wing membrane biopsies (3 mm) were collected according to Worthington‐Wilmer and Barratt^45^ from all captured bats. The samples were kept in 96% ethanol at − 20 °C until processed in the laboratory for molecular analysis to confirm species identification (see^41^ for laboratory procedures).

Wing morphology variables were measured with a calliper and included the lengths of the forearm (FA), metacarpals of the III and V fingers and the first phalanges of the same fingers, as well as the length of the upper dental series (canine to the third molar—CM3) and the rostral width measured at the canines’ level (C1–C1).

Coat colour was quantified by measuring the reflectance through a High-Resolution Colorimeter (Spectrophotometer CM-2600d/2500d, Konica, Minolta). Two measurements were obtained for dorsal and ventral colours from each bat. Each measurement was the average of three light flashes’ reflectance. Reflectance was measured at a wavelength band from 350 to 740 nm and was decomposed according to the colour space defined by CIELAB colour space, which included three parameters, L*, a* and b*. L* represents perceptual lightness, and a* and b* a four colours scale, from red to green and blue to yellow, respectively (Supplementary Fig. S2)^46^.

To study the effect of interspecific responses of *E. serotinus* and *E. isabellinus* and their hybrids, bats were clustered into five experimental groups: allopatric *E. isabellinus*, sympatric *E. isabellinus*, hybrids (individuals classified as hybrids by Centeno-Cuadros et al.^41^ based on genetic data, using conservative criteria), sympatric *E. serotinus* and allopatric *E. serotinus*. We assumed that both species were geographically isolated from each other and the respective hybrids within the allopatric groups. On the contrary, in the sympatric groups, each species co-existed with the other species and their hybrids. The geographic classifications of each experimental group were based on previous studies^37,41,42^.

### Statistical analyses

#### The effect of geographic origin and hybrids’ presence on bat morphometry

We used General Linear Models to assess whether geographic relationships significantly affected the morphometry and/or the colour of the studied bats. We performed a non-parametric MANOVA^47^ on the morphological variables because the homogeneity of variance could not be assumed according to the Box’s M tests (Morphometry: Box’s M = 133.73; F_(84, 16828.68)_ = 1.393; ns; Colour: Box’s M = 215.69; F_(63, 1824.12)_ = 2.480; ns) even after trying several data transformations (log_10_, ln, arcsen, etc.). We performed a Kruskal–Wallis test to identify which morphometric traits and colour variables were significantly different, followed by multiple comparisons by the post-hoc Fisher’s LSD test to check for differences between groups^47^.

#### Summarizing and comparing morphological variables

The original morphometric space of the wing and dentition variables was summarized into its main components by a principal component analysis (PCA) with the symmetrical normalization method^47^. The eigenvalue rule greater than 1 was used as the criterion for components’ retention alongside the scree-plot. We analysed the internal consistency of each component through Cronbach's alpha^47^.

After the validation of both assumptions for multivariate normal distribution and homogeneity of variance–covariance (M = 13.98; F_(12, 7934.55)_ = 1.104, *p* = 0.352), we performed a MANOVA over the PCA’s axes to evaluate the relationship of the five experimental groups with morphometric traits. When the MANOVA detected significant effects, we performed an ANOVA for each component (PC1 and PC2), followed by multiple comparisons through Tukey's HSD post-hoc test. Again, according to the Box’s M tests, even after transformation, the homogeneity of covariance could not be assumed (Box’s M = 30.41; F_(12, 2323.67)_ = 2.250; ns) for retained colour’s PCA. To assess whether geographic origin affected colour variables, we performed a non-parametric MANOVA^47^, followed by the tests described above^47^. We carried out all analyses with α = 0.05 in SPSS Statistics software (v. 22; SPSS Inc, Chicago, IL; https://www.jmp.com/), producing all visualizations through the “ggplot2” R package^48^.

#### Classification statistics with and without hybrids’ presence

We used two classification algorithms to classify both species in sympatry according to the morphological variables: discriminant function analysis (DFA—quadratic with cross-validation) and the support vector machines (SVM—with a machine learning approach).

The morphometric matrices fulfil the assumptions of normal distribution and homogeneity of variance so we proceeded with parametric analyses (Morphometry with hybrids: Box’s M = 38.33; F_(28, 32463.13)_ = 1.267; *p* = 0.157; Morphometry without hybrids: Box’s M = 11.10; F_(10, 5819.66)_ = 0.996; *p* = 0.444).

The data were firstly included in two stepwise discriminant analyses with the method of Wilks’ Λ used to identify which of the morphological and colour variables under study can better discriminate both parental species with and without the presence of hybrids. Again, homogeneity of the variance–covariance matrices could not be assumed for the colour variables (Colour with hybrids: Box’s M = 37.65, F_(10, 11071.13)_ = 3.474, ns; Colour without hybrids: Box’s M = 38.06, F_(10, 4588.83)_ = 3.414, ns). Still, we proceeded with analysis because the discriminant analysis is robust to violation of assumptions when (i) the size of the smallest group (sympatric *E. serotinus*) is greater than the number of variables in the study and (ii) the means of the groups are not proportional to their variances^47^. Finally, we used Classification Statistics to obtain the classification functions and to assign species identification according to morphometric traits and/or colour variables. All DFA analyses were carried out with α = 0.05.

In addition, a support vector machine (SVM) algorithm was performed using the principal components extracted from each PCA (morphometry and colour), through “tidyverse” (performing data manipulation)^48^, “kernlab”^49^ and “e1071” R packages^50^ (performing calculations and producing visualizations). The kernel functions of the support vector machines used a radial basis function with a gamma parameter ranging from 0.1, 1, 10, 100 and 1000. For each value of gamma, the SVM was reinitialised 20-times to increase the chance of obtaining an optimal classifier^51^. For Classification Statistics the algorithm of the SVM was trained for every target case in the dataset (each species)^51^. All classifiers were then combined and categorised for each morphological trait as either belonging to a specific class or not. Each bat was considered as classified correctly only if a single support vector machine classified it and if validated by molecular identification.

#### Quantification of the impact of the hybrids on the external morphological traits overlap between species

We repeated the entire statistical procedure after extracting from the dataset all individuals classified as hybrids in Centeno-Cuadros et al.^41^. We compared the classification performance of both algorithms in the sympatric population with hybrids versus the same population after excluding them. This approach made it possible to calculate the contribution of hybrids to the overlap of morphological traits with their cryptic parental species, based on the percentage of hybrids among the misclassified individuals through the classification statistics.

## Results

### Testing for morphological differences between cryptic *Eptesicus* species

Our results showed significant differences in both morphometric and colour variables along the experimental groups. Morphometric differences occurred for the variables FA, D3 MC, D5 MC, D5 F1, C1-C1 and CM3 but not for the variable D3 F1 (Table 1).

**Table 1:** Descriptive statistics of the morphometric traits of *Eptesicus serotinus*, *E. isabellinus* and their hybrids.

	Wing					Dentition	
	FA	D3 MC	D3 F1	D5 MC	D5 F1	C1-C1	CM3
*E. isabellinus* allopatric (Eisa allo; n = 31 individuals)	51.6	47.1	17.8	44.1	10.9	6.7	7.2
	*49.9–55.7*	*44.0–52.0*	*12.2–19.8*	*41.3–48.0*	*10.0–12.0*	*6.3–7.2*	*6.8–7.7*
*E. isabellinus* sympatric (Eisa sym; n = 33 individuals)	51.6	46.1	18.0	43.0	11.3	6.9	7.2
	*48.6–54.3*	*42.6–48.6*	*16.5–20.5*	*40.1–48.5*	*10.2–12.6*	*6.3–7.4*	*6.5–8.4*
*Hybrids* (n = 8 individuals)	52.6	46.8	18.8	43.4	12.0	6.7	7.4
	*49.7–53.7*	*42.7–47.7*	*16.8–19.1*	*39.9–45.9*	*11.0–13.3*	*6.5–6.9*	*7.1–7.5*
*E. serotinus sympatric* (Eser sym; n = 19 individuals)	52.8	47.5	17.8	44.5	11.7	6.9	7.6
	*49.7–55.2*	*44.7–49.7*	*16.8–20.4*	*40.5–47.2*	*9.9–13.1*	*6.6–7.5*	*7.3–8.5*
*E. serotinus allopatric* (Eser allo; n = 35 individuals)	52.6	49.0	18.0	46.5	11.3	6.9	7.7
	*48.8–56.1*	*41.4–51.6*	*13.2–19.5*	*41.6–49.6*	*10.0–12.5*	*6.6–7.5*	*7.4–8.7*
*X*^*2*^_*KS*_ (4)	*13.302*	*38.271*	*1.698*	*25.234*	*12.244*	*15.300*	*64.410*
*p*-value	*0.010*	< *0.001*	*ns*	< *0.001*	*0.016*	*0.004*	< *0.001*

*Medians are shown above the range.*

**Figure 1:**
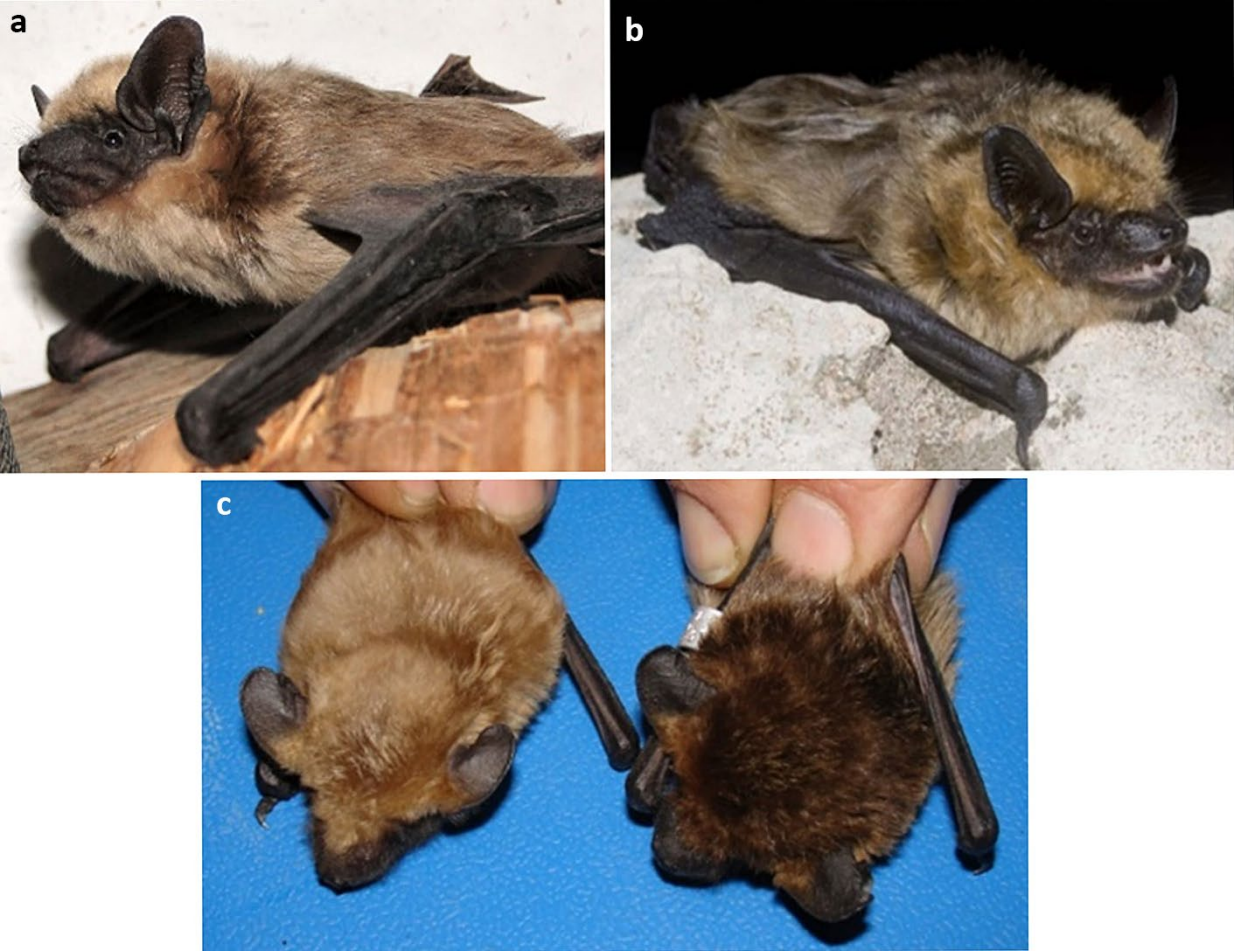
Colour differences between bat groups. Photographic representation of hair colour of representative specimens of both parental species groups: (**a**) allopatric *Eptesicus isabellinus,* (**b**) allopatric *E. serotinus* and (**c**) sympatry *E. isabellinus*— on the left - and sympatric *E. serotinus* - on the right.

For coat colour, we found significant differences between the groups in the variables L* dorsal, a* dorsal, b* dorsal, a* ventral and b* ventral, but not for the variable L* ventral (Table 2). Photographic representation of hair colour of representative specimens of both parental species groups is available in Fig. 1.

**Table 2:**
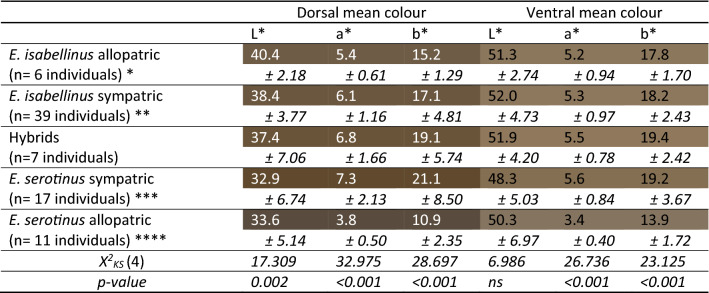
Descriptive statistics of the dorsal and ventral colour of *E. serotinus*, *E. isabellinus* and their hybrids.

Background colour represents the mean colour of the dorsum and belly in the CIELAB colour space of each parental species in function to their different geographical relationships and their hybrids.

**Figure 2:**
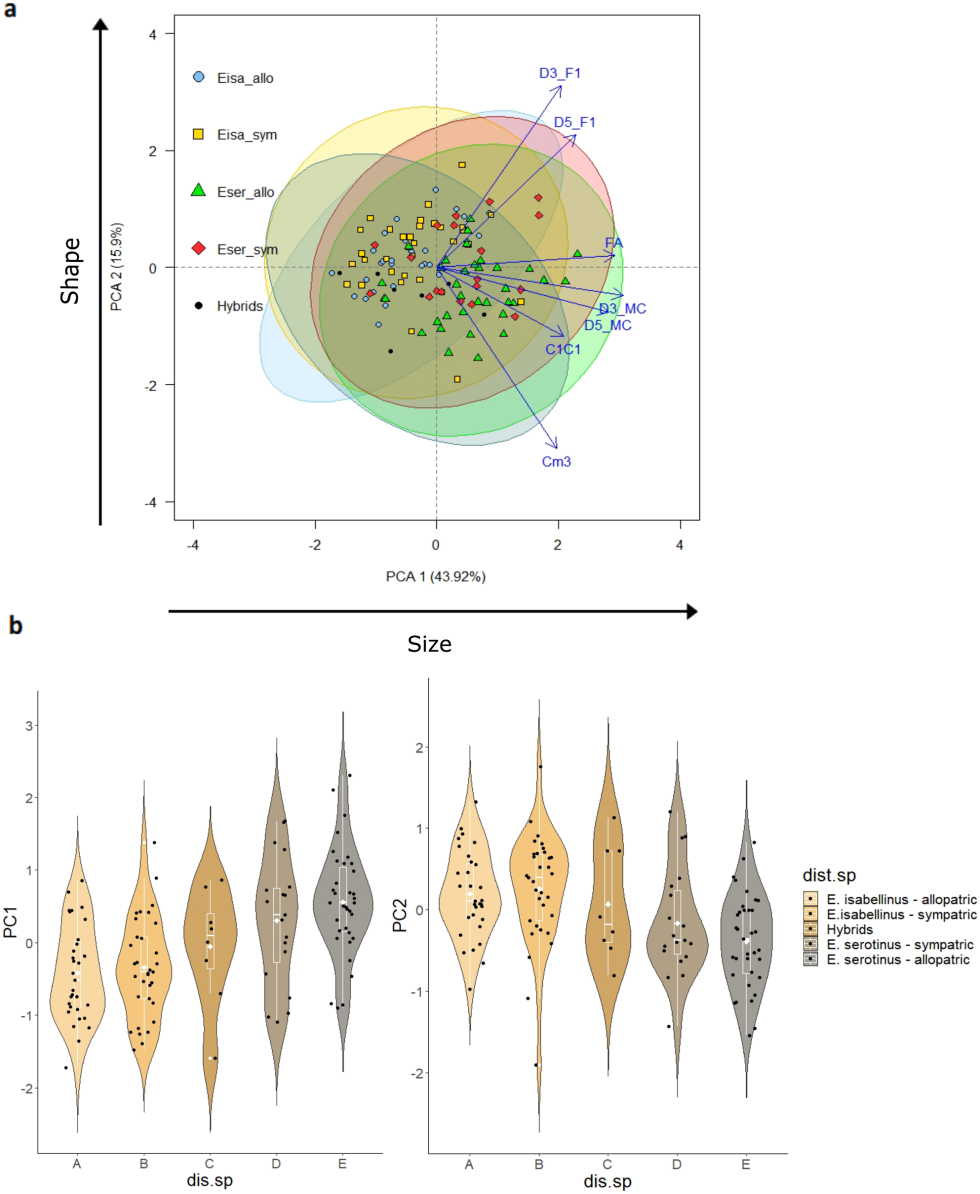
Principal Component Analyses of morphometric variables. Positioning of each individual in the two-dimensional space defined by the main PCA components retained and their position relative to the original morphometric variables (**a**). The violin plots represent *E. isabellinus* (allopatric and sympatric), *E. serotinus* (allopatric and sympatric) and Hybrids PCA scores compared to size (PC1) and shape (PC2) (**b**). Within violin plots, white solid lines boxplots include medians (horizontal white line) and averages (white dot); boxes and vertical white lines indicate quartiles and ranges (excluding outliers), respectively. Acronyms are available in Table 1 (dist.sp: species distribution).

The information between both morphometric and colour variables was summarized in two main orthogonal components, explaining respectively 59.8% and 83.4% of the total variance of the original morphometric and colour variables (see Supplementary Table S2). The first morphometric component was associated with bats’ “Size”, whereby generally, the weight of all variables was very high and positive. The second morphometric component essentially summarized the wing "Shape", namely the metacarpals and first phalanges proportion. Additionally, the inversely proportional relationship between the length of the first phalanges and the dentition variables, mainly CM3, had a very high and negative score in this second component (Supplementary Table S2). Despite an evident overlap, the two-dimensional PCA map showed that *E. serotinus* is generally larger in both wing and dentition, particularly in the allopatric zone concerning the *E. isabellinus*. In sympatry, there was a greater overlap between both species despite maintaining the same pattern (Fig. 2).

*Eptesicus isabellinus* both in allopatry and sympatry showed a greater proportion between the first phalanges and both metacarpals (e.g. smaller ratio of the length of both metacarpals to the first phalange), revealing an inversely proportional relationship between the length of the first phalanges and both dentition variables (larger phalanges and smaller C1-C1 and mainly CM3) (Fig. 2). In contrast, *E. serotinus* revealed, in both sympatry and allopatry, to have substantially smaller first phalanges relative to metacarpals, as well as to both dentition variables (mainly CM3) (Fig. 2).

**Figure 3:**
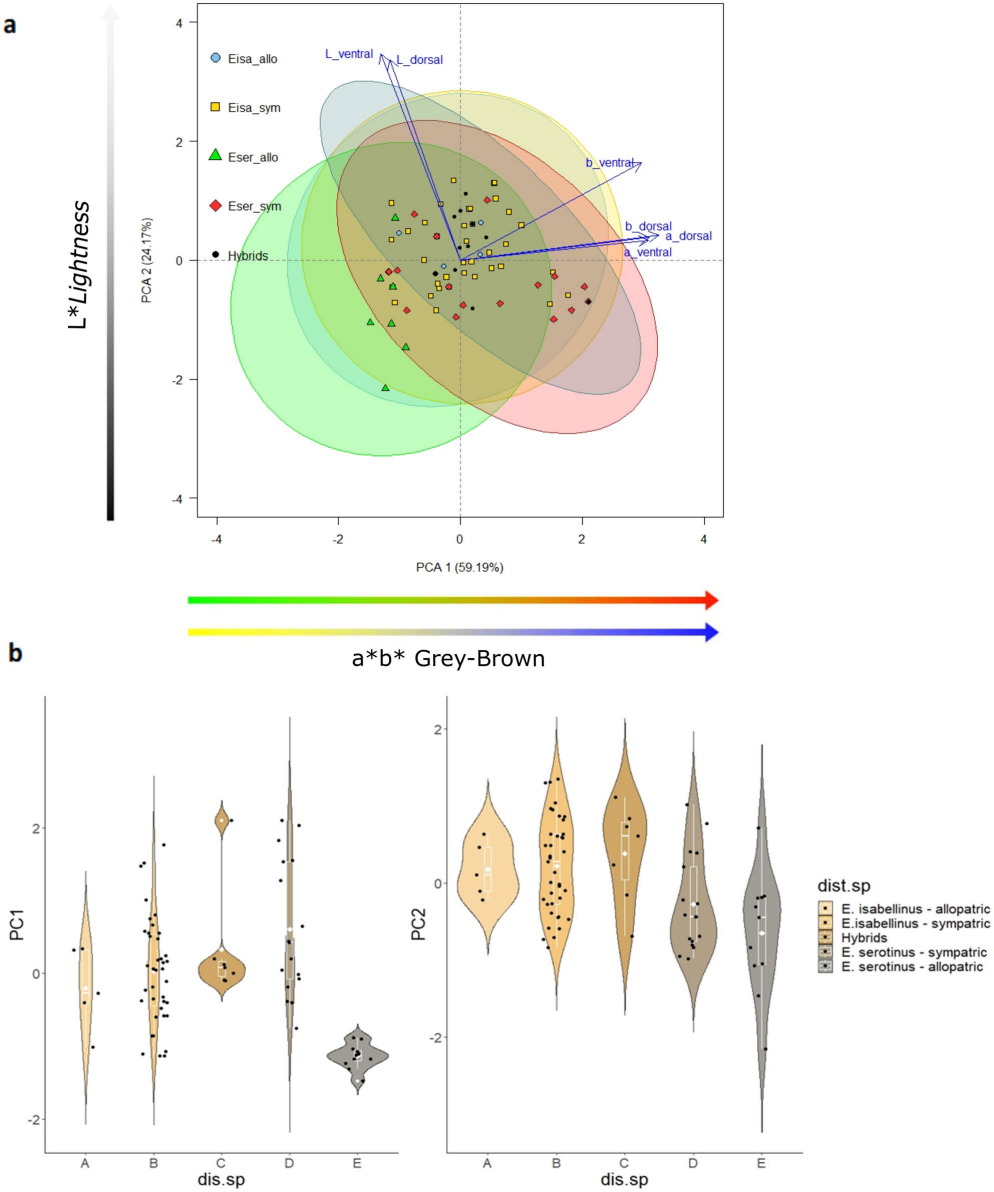
Principal Component Analyses of colour variables. Positioning of each individual in the two-dimensional space defined by the PCA main components retained and their position relative to the original dorsal colour variables (**a**). The violin plots represent *E. isabellinus* (allopatric and sympatric), *E. serotinus* (allopatric and sympatric) and Hybrids PCA scores compared to dorsal a*b* Grey-Brown (PC1 Colour) and dorsal L* lightness (PC2 Colour) (**b**). Within violin plots, white solid lines boxplots include medians (horizontal white line) and averages (white dot); boxes and vertical white lines indicate quartiles and ranges (excluding outliers), respectively. Acronyms are available in Table 1 (dist.sp: species distribution).

The first colour component of the PCA was associated with a range of colours from grey to brown, named as “a*b* Grey-Brown scale”, while the second component essentially summarized the “L*Brightness” (Table S2). Despite an evident overlap, it was possible to characterize allopatric *E. serotinus* as being darker and greyer by showing a combination of higher black, green and yellow components in both dorsal and ventral colourations (Fig. 3). All the remaining groups varied mainly along the brown pallet by having higher values for red and blue. Sympatric *E. serotinus* was in the other extreme of that Grey-Brown colour pallet, being the brownest group (Supplementary Table S4). Likewise, sympatric *E. serotinus* shared the darkest position with allopatric *E. serotinus*, on the brightness scale (“L* brightness”). Allopatric *E. isabellinus* was the brightest group. In sympatry, once again, *E. isabellinus* showed the brightest colour patterns. The ventral colour was always brighter than the dorsal one in all experimental groups due to its higher L* values, showing even more subtle colour differences between groups (Fig. 3).

#### Sympatry versus allopatry: influence on the morphology

There were no significant differences in the bats’ size and shape between allopatric and sympatric groups within each species (except in metacarpals). However, all morphological variables differed significantly between allopatric groups. Regarding morphometric variables (D5 MC, D5 F1 and C1C1), especially for bats’ “shape”, these significant differences disappear in sympatry (a detailed description of the morphometric comparisons is available in Supplementary Material).

Regarding the colour variables, all experimental groups differed significantly from allopatric *E. serotinus* in the a* b* Grey-Brown scale, which included the only individuals in the grey range of the colour pallet. In terms of brightness, allopatric *E. serotinus* was only not significantly different from individuals of the same species when in sympatry (bats were equally dark, though in sympatry they tended to be significantly browner). In sympatry, both species also differed significantly, however, contrary to the morphometric variables, this difference was slightly greater than that found between species in allopatry.

#### Hybrids versus parental species: the impact of hybridization on morphology

Hybrids did not differ significantly from the other groups of allopatry/sympatry in wing size (except for metacarpals), showing intermediate wing morphometric values relative to both parental species. Hybrids differed significantly in the dentition between the two species, even in comparison with both sympatric groups with which they coexist (e.g. CM3). Nonetheless, hybrids showed consistently intermediate phenotypes in both dentition and wing size, as well as wing shape (a detailed description of the morphometric comparisons between hybrids and parental species is available in the Supplementary Material).

Hybrids showed the brightest pelage colour (both in their dorsal and ventral coat), although not significantly different from *E. isabellinus* in both groups (sympatry and allopatry) (Fig. 3; Supplementary Table S5). On the other hand, hybrids were significantly browner than allopatric *E. serotinus* and they were also the brownest group together with sympatric *E. serotinus* (Supplementary Table S5).

### Species discrimination through classification statistics

#### Stepwise DFA with and without hybrids

The morphometric stepwise DFA generated one discriminant function mainly defined by D3_MC and CM3 (Λ = 0.579, χ^2^_(7)_ = 53.773, *p* < 0.001). The percentage of individuals classified correctly in the presence of hybrids was 81.7%. After hybrids removing, the discriminant analysis generated also one discriminant function, with FA, D3_MC, D5_MC (marginally significant [F = 3.450, *p* = 0.069]) and CM3 as statistically significant variables (Λ = 0.632, χ^2^_(4)_ = 21.073, *p* < 0.001). The results of Classification Statistics showed that the total percentage of individuals classified correctly increased to 84.0% after hybrids removing.

The colour stepwise DFA generated also only one discriminant function, with L*_dorsal, a*_dorsal, b*_dorsal and L*_ventral as statistically significant variables (Λ = 0.842, χ^2^_(4)_ = 10.115, *p* = 0.039). The percentage of individuals classified correctly in the presence of hybrids was 71.4%. After removing the hybrids, the colour discriminant analysis generated also one discriminant function, once again with L*_dorsal, a*_dorsal, b*_dorsal and L*_ventral as statistically significant (Λ = 0.762, χ^2^_(4)_ = 14.114, *p* = 0.007). The results of Classification Statistics showed that the total percentage of individuals classified correctly increased to 78.6% after hybrids removing.

#### Support vector machine with and without hybrids

SVM generated one discriminant function based on morphometric PC1 and PC2 as predictors’ variables that summarized all morphometric traits. After simulations, the results of Classification Statistics showed that the percentage of individuals classified correctly in the presence of hybrids was 75.0%. Without hybrids, SVM generated also one discriminant function. The percentage of individuals classified correctly increased to 76.9% after removing hybrids.

Based on colour PC1 and PC2 as predictors’ variables, SVM generated also one discriminant function. After simulations, the results of Classification Statistics showed that the percentage of individuals classified correctly in the presence of hybrids was 68.3%. After hybrids removing, the colour SVM also generated one discriminant function. The results of Classification Statistics showed that the total percentage of individuals classified correctly increased to 76.8% after removing hybrids (a detailed description is available in Supplementary Material).

Finally, both species showed a statistically significant differential behaviour concerning the correct classification rate for both classification methods tested (χ^2^_*kW*_ (1) = 10.678, *p* = 0.01, N = 16) and *E. isabellinus* individuals were significantly classified more correctly than the individuals of *E. serotinus* (F = 15.976; *p* = 0.001) (Table 3).

**Table 3:** Correct species classification rates, species overlap and hybrids’ contribution to species overlap that were obtained by two different methods of classification statistics (DFA and SVM) based on morphometric and colour traits of *E. isabellinus* and *E. serotinus* in sympatry.

	DFA (%)	SVM (%)
**Morphometry**		
*E. isabellinus* correct classification rate	84.5	78.9
*E. serotinus* correct classification rate	78.3	72.3
*E. isabellinus* correct classification rate without hybrids presence	96.0	83.9
*E. serotinus* correct classification rate without hybrids presence	66.7	66.7
The global correct classification rate	81.7	75.0
Global correct classification rate without hybrids presence	84.0	76.9
Overlap in sympatry	18.3	25.0
Hybrids’ contribution to species overlap	13.6	7.6
**Colour**		
*E. isabellinus* correct classification rate	92.3	92.3
*E. serotinus* correct classification rate	37.5	29.2
*E. isabellinus* correct classification rate without hybrids presence	92.3	77.1
*E. serotinus* correct classification rate without hybrids presence	47.1	75.0
The global correct classification rate	71.4	68.3
Global correct classification without hybrids presence	78.6	76.8
Overlap in sympatry	28.6	31.7
Hybrids’ contribution to species overlap	25.2	26.8

### General impact of introgressive hybridization on morphological overlap of cryptic *Eptesicus* bats

Based on the two tested algorithms and the two sets of traits (morphometry and colour), the overlap between the two parental species was around 25.9 ± 5.76%. The contribution of hybrids to overlap between parental species was significantly higher for colour (26.0 ± 1.13%) than morphometry (10.6 ± 4.24%) (F = 24.602; *p* = 0.038). Overall, hybrids contributed on average 18.3 ± 9.25% to traits overlapping between their parental species (Table 3).

## Discussion

Differences in morphology have been commonly used to differentiate taxa undergoing preliminary steps of macroevolution. However, as predicted by the particular challenges posed by the similarity of cryptic species, overlap in all morphological variables was the most evident pattern. Yet, for the two species of Iberian *Eptesicus,* the differences in size and shape were strikingly larger when comparing allopatric populations than when comparing populations in sympatry. Still, the high overlap in all morphological traits meant that no single variable could be used as a diagnostic characteristic to distinguish the two species. Even so, the analyses of the external morphological traits (wing and dentition) confirmed that *E. serotinus* is slightly, but significantly, larger and darker than *E. isabellinus*. Although allopatric *E. serotinus* bats showed a greyer coat, the remaining four groups were distributed along the brown pallet, with sympatric groups showing the brownest coat. Molecularly confirmed hybrids showed intermediate morphometric values between both parental species in terms of size and shape, particularly when compared to allopatric populations. Hybrids were significantly browner than allopatric *E. serotinus*, showing the brightest coats of all, even brighter than both allopatric and sympatric *E. isabellinus*. Thus, alongside a probable process of adaptation to the local environmental conditions in the sympatric zone, bidirectional adaptive introgression (even if asymmetrical) can lead to convergence in size and shape, increasing morphological overlap among parental species^4^. Hybrids, distributed along a sympatric zone, show intermediate values in morphometric traits (both in size and shape), while differences between both parental species in morphometry decreased significantly in sympatry. Therefore, it is probable that hybridization and adaptive introgression affect the morphology of the parental species through backcrossing^9,18^.

External traits are priority targets for natural selection as they directly impact the fitness of individuals and the ecological niche in which they occur^52^. According to Grant and Grant^4^, it is possible to make genetic inferences grounded in two morphological aspects, size and shape, due to the narrow connection between introgressed alleles and species morphology. This fact is based on the high frequency of heritable variation and polygenic origin of external traits^53^. This relationship is supported by evidence from genes that were identified as regulating the development of vertebrates’ size and shape^54^. There is a considerable amount of evidence that supports the influence of adaptive introgression on morphometry impacting both size and shape^18,55^. Adaptive introgression consequences seem plastic enough to be able to promote divergent adaptations in response to the particularities of environmental conditions^9^. For example, adaptive introgression events promoted size reduction in ancient humans, allowing them to adapt to insular environments^20^, while on the other hand, they enabled carnivores to obtain larger prey by increasing their body size^55^.

Adaptive introgression of bat morphological traits should respond to the circumstances in which they co-occur within the sympatric zone. Adaptive introgression in *Eptesicus* dentition and wing morphology, for example, could impact their trophic space, being connected to their diet, as well as their ecological niches, responding directly to the macro-habitat characteristics of the sympatric zone and local prey availability. With increasing dentition size, *E. isabellinus* is more able to capture larger prey, while the smaller dentition of *E. serotinus* makes it more efficient in capturing smaller prey^56^. On the other hand, increased wing size and shape changes (namely decreasing the proportion between first phalanges and metacarpals) may have enabled *E. isabellinus* to become more efficient in exploring more open habitats^57^ and disperse over longer distances^58^. In contrast, decreased wing length and shape changes (namely increasing the proportion between first phalanges and metacarpals) may have rendered *E. serotinus* more prone to explore close habitats than those used in allopatry^57^.

Mammals are not frequently colourful being mostly colour-blind to the red-green spectrum because they tend to be crepuscular or nocturnal^59^. Bats are an extreme example of it. Under low-light conditions, dichromatic vision seems to be advantageous over colour vision because the reduced number of colour-sensitive cones in the retina means they focus on more light-sensitive rods, thus improving their visual acuity^60^. Similar to other nocturnal mammals, bats are commonly in shades of black, grey or brown, with only small colour differences between species^61^. Thus, the differences found in bat colours are substantially smaller than morphometric ones. Still, contrary to the patterns found in morphometry, the colour characteristics of hybrids are at the extreme of the brown colour palette. In parallel, they are also consistently the brightest ones. Still, the differences in colour parameters of both parental species seem slightly greater in sympatry.

Colour patterns have been linked to communication and physiological processes, such as thermoregulation^62^. However, most bats use vocal and olfactory cues rather than visual signals to perceive the environment and for social interactions^63^. On the other hand, brighter colours cannot affect mating recognition without the development of tri- or tetra-chromatic vision^62^. Bat colouration is most probably the result of adaptation to a nocturnal niche, likely associated with concealment from predators^62^. So, bats’ colouration should reflect environmental pressures, for example, from the roosts that they occupy. Different colour patterns can be expected in species occupying different types of roosts because roost conditions differ in their visual environment (e.g. luminance and colour spectrum), as well as in their level of exposure to twilight or diurnal predators^62^.

The two Iberian *Eptesicus* species do not share roosts, despite their colonial behaviour with nursery colonies comprising up to 300 animals^64^, and their tendency to share roosts with other species^65^. This suggests that these two bats may compete actively for the best spots when in sympatry. *Eptesicus serotinus* colonies are mainly associated with buildings in Central Europe^66^, while in the Mediterranean region, they also roost in crevices of cliffs and at the entrance of caves^64^. *Eptesicus isabellinus*, instead, seems to prefer crevices (cliffs or bridges) and tree hollows in date palms in North Africa^67^.

Resource partitioning and geographic avoidance have been described for several pairs of cryptic bat species across Europe^43,68^. Cryptic species can exploit different microhabitats and roosts to avoid competition when sharing the same sympatric area^68^, and this may be the case for the Iberian *Eptesicus* in sympatry. Therefore, roost-mediated coevolution could be one plausible hypothesis to explain the different colour patterns in bats. The light environment of nocturnal niches can be dominated by yellow-green wavelengths, for example in more closed foraging spots, which coincides with the spectral sensitivity of bats^69^. Therefore, the natural pressure of nocturnal light conditions occurring over the various microhabitats that are explored by each species in sympatry may have interacted with diurnal light conditions in roosts to favour the pelage colour differences in *Eptesicus*. Thus, any possible differences promoted by the interactions between the two cryptic bats in sympatry through potential competition for roosts and microhabitats will not only have consequences for their colour and their discrimination but fundamentally for their interactions with predators and the environment^70^.

The extent of morphological overlap between the two cryptic bats was around 26%. The presence of hybrids contributes substantially to a fifth of the total traits overlap. Even so, classification statistics showed a high differentiation rate between the two parental species in sympatry, particularly for *E. isabellinus*, with higher correct identification rates for morphometric traits, which were more distinct than colour.

Discriminant functions showed that D3_MC and CM3 are the main variables discriminating between the species. However, only CM3 showed limited overlap between them with values below 7.3 cm found only in *E. isabellinus*, whereas values above 8.4 cm only in *E. serotinus.* Therefore CM3 is the only measure that can be used to confidently distinguish between the two species.

Morphological diversity is a critical facet of evolution and adaptation^71^. However, morphology is also extremely important for field identification, particularly for endangered species. Accurate field identification is particularly important for *Eptesicus* species because they are hosts of different lineages of the European bat Lyssavirus type 1 (EBLV‐1), the most common rabies-related virus found in European bats^72,73^.

### Caveats and limitations

We classified individuals as hybrids following Centeno-Cuadros et al.^41^ and applying the conservative criteria proposed by Burgarella et al.^74^ to the microsatellite genotypes to guarantee there were no misclassifications. To be classified as hybrids, individuals had to meet two criteria: (a) The sum of q‐values must be higher than 0.75 for all hybrid categories in NEWHYBRIDS software (v. 1.1 beta; http://ib.berkeley.edu/labs/slatkin/eriq/software/software.htm) and (b) must be assigned to one of the two *Eptesicus* species with a q-value lower than 0.90 in STRUCTURE software (v. 2.3; https://web.stanford.edu/group/pritchardlab/software.html)^41^. However, this approach does not allow us to exclude the possibility of the presence of other possible hybrids (who did not meet some of these criteria) in the other sympatric groups. In addition, the lab methodology used to detect hybrids does not detect the presence of second-generation and later hybrids, as well as backcrosses. These potentially overlooked hybrids may be falsely inflating the overlap between morphological characteristics of the two sibling bat species in sympatry. Despite this, we still obtained high values of correct identifications in the classification statistics, which indicates that the proportion of undetected hybrids was low.

## Conclusions

We demonstrated that cryptic Iberian *Eptesicus* species can coexist during secondary contacts under antagonistic evolutionary forces, imposed simultaneously by introgressive hybridization and potential niche-partitioning. This balance may be driven by the partitioning of resources along two niche axes, microhabitats and roosts. The situation may be different in other niche axes, such as trophic resources, prey characteristics and macro-habitats, which are under adaptive pressure from local conditions. Divergence due to niche differentiation and adaptive introgression should impact different aspects of morphology^75^. The latter seems to have a greater effect on the divergence of bat pelage colour. Instead, adaptive introgression seems to act as a homogenising force over morphometric traits, such as wing and dentition shape and size. Finally, we argue that a balance between adaptive introgression and interspecific competition, beyond mediating species divergence, shapes their interactions with the environment by impacting morphological traits under selection.

## Supplementary Information

Supplementary Information.

## Data Availability

Data generated and analysed during this study are included in this published article (and its Supplementary Information files) as well as in the supporting information from previous publications^41^. Additional data and information are available from the corresponding author on reasonable request.
