# Supplementary material for: Counteracting forces of introgressive hybridization and interspecific competition shape the morphological traits of cryptic Iberian Eptesicus bats
Source: Sci Rep. 2022 Jul 8;12:11695. doi: 10.1038/s41598-022-15412-2 (PMC9270368; doi:10.1038/s41598-022-15412-2)
Supplement: Supplementary file 1 — Supplementary Information. [file 41598_2022_15412_MOESM1_ESM.docx]

Supplementary Materials for

**Counteracting forces of introgressive hybridization and interspecific competition shape the morphological traits of cryptic Iberian *Eptesicus* bats**

Pedro Horta*, Helena Raposeira, Adrián Baños, Carlos Ibáñez, Orly Razgour, Hugo Rebelo, Javier Juste

*Corresponding author: [pedro.horta@cibio.up.pt](mailto:pedro.horta@cibio.up.pt)

**This file includes:**

Supplementary Text

Figs. S1 to S3

Tables S1 to S5

**
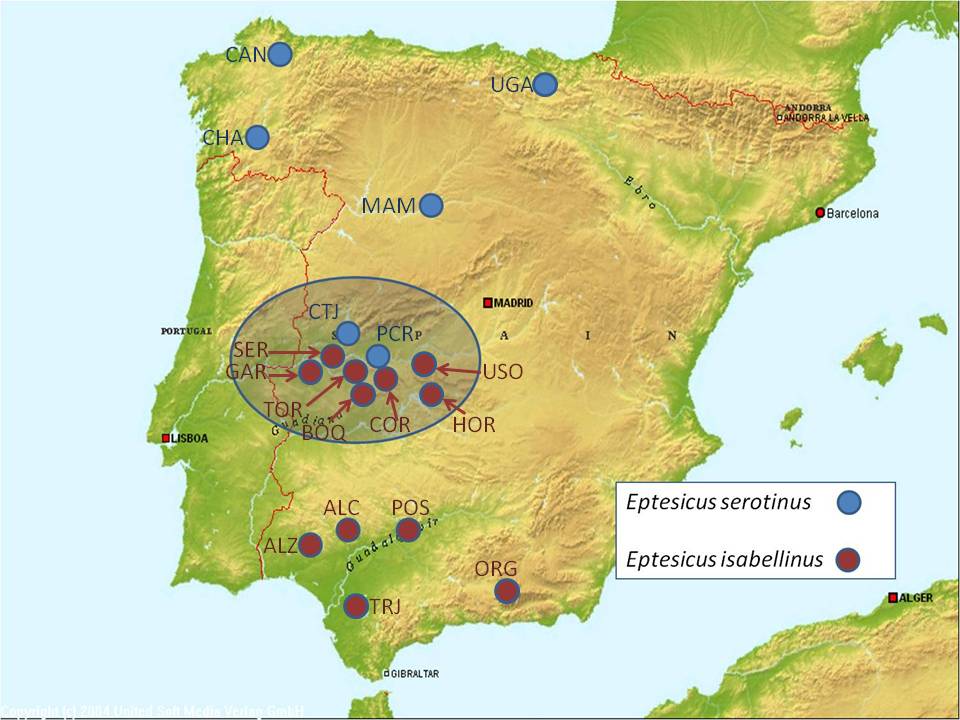
**

**Figure S1.** Sampled colonies of *Eptesicus serotinus* (blue dots) and *E. isabellinus* (red dots) in the Iberian Peninsula. The sympatric zone is marked with a shaded ellipse. CAN O Caneiro (A Coruña); CHA Chaín (Pontevedra); TUD Tudela del Duero (Valladolid); UGA Ugao (Vizcaya); CTJ Casatejada (Cáceres); PCR Pozo del Rey (Cáceres); ALC Alcalá del Río (Sevilla); AZN Aznalcollar (Sevilla); ORG Órgiva (Granada); POS Posadas (Córdoba); TRJ Puente Trajano (Sevilla); HOR Horcajo de los Montes (Ciudad Real); USO Puente Río Uso (Toledo); BOQ Boquerón (Ciudad Real); COR Corrinche (Cáceres); GAR Garrovillas (Cáceres); JAR Jaraicejo (Cáceres); SER Serradilla (Cáceres); TOR Torrejón el Rubio (Cáceres). Figure generated through QGIS software (v. 3.24.2, <https://qgis.org/en/site/>).

**
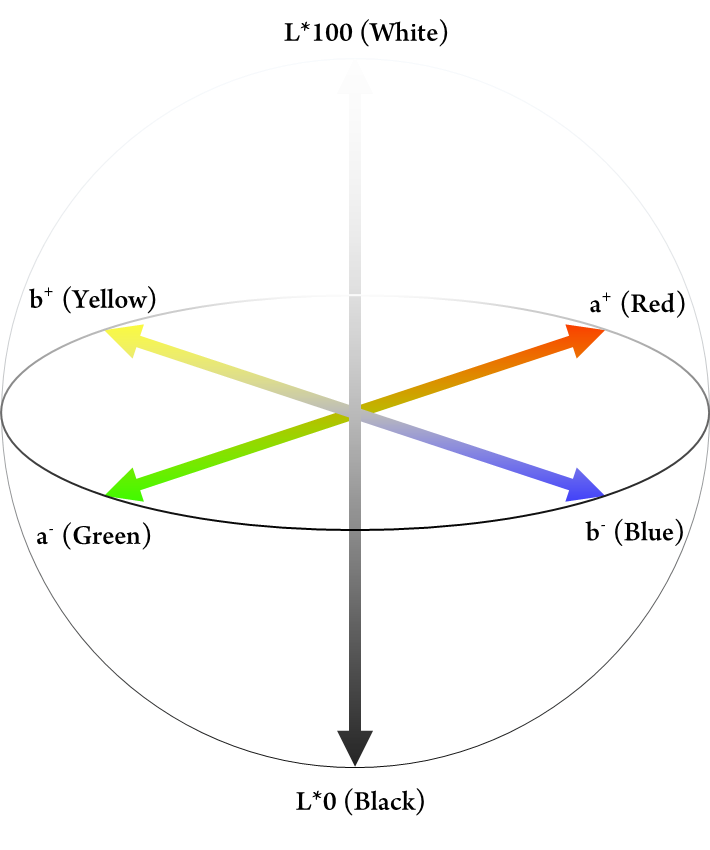
**

**Figure S2.** CIELAB colour space expressing colours in three values: L* for perceptual lightness, and a* and b* for the scales of the four colours of human vision: red and green, blue and yellow, respectively. Figure generated through Inkscape software (V.1.1.2; <https://inkscape.org/>).

**Multiple comparisons of morphometric variables**

**Allopatry vs sympatry**

Within each species, there were no differences in forearm (FA) length between allopatric and sympatric groups, though both groups of both species differed significantly from the others of its sibling. Nevertheless, FA differences between the two species in allopatry were slightly higher than that found in sympatry.

Allopatric and sympatric groups differed significantly in the variable D3 MC within each species. The same also occurred between sympatric groups of species, as well as between their allopatric groups. Differences between both species in allopatry were also slightly higher than that found in sympatry. Multiple comparisons in D5 MC followed the same pattern. Allopatric and sympatric groups differed significantly within each species. However, there were no significant differences between species in the sympatric area, only between individuals of allopatric regions.

For phalange measures, such as D5 F1, all groups differed from allopatric *E. isabellinus* due to its lowest length, except for individuals living in sympatry with *E. serotinus*. Therefore, in allopatry, both species differed significantly in the size of the first phalanges, while in sympatry there were no significant differences.

There were no differences in the dentition variable CM3 within species between allopatric and sympatric groups. However, both groups of both species differed significantly from the other species. In this case, differences in CM3 between both species in allopatry were essentially similar than that found in sympatry.

Finally, the results demonstrated statistically significant differences in the C1-C1 variable between allopatric *E. isabellinus* and both groups of *E. serotinus* (sympatric and allopatric). However, in that case, under sympatry, there were no significant differences between both species unlike what happens in allopatry.

From a multivariate point of view, there were no significant differences in the bats’ size between allopatric and sympatric groups within each species. However, both groups of both species differed significantly from the other species’ groups. Nevertheless, size differences between both species in allopatry were slightly higher than that found in sympatry. Regarding the shape component (PC2 morphology), there were no significant differences within each species when in the presence or absence of its sibling. No significant differences were found among individuals of the two species when they were in sympatry, while in allopatry the differences in the bats’ shape were significant. For allopatric *E. serotinus* the shape component stood out as substantially different, making it significantly different even from the sympatric *E. serotinus* group.

**Hybrids vs parental species**

The FA length of hybrids did not differ significantly between groups, with both parental species showing intermediate values. *E. serotinus* had the longest average length of FA (both allopatric and sympatric, in this order) followed by the hybrids and *E. isabellinus* (sympatric and allopatric).

Regarding the metacarpals, hybrids only differed significantly from allopatric *E. serotinus,* having significantly lower D3 MC and D5 MC. Allopatric *E. serotinus* had the longest metacarpals and hybrids showed the lowest values in both fingers (D3 and D5). Sympatric groups revealed intermediate values between the respective allopatric groups of each species and the hybrids (Table 1). The exception was the sympatric *E. isabellinus* group which shared the lowest values with hybrids in the metacarpals of the fifth finger.

In contrast, hybrids had the longest first phalanges although they did not differ from the rest of the groups. Both sympatric groups had higher values than the respective allopatric groups and intermediate between them and the hybrids (Table 1).

Hybrids showed intermediate values of CM3 between both parental species, with significantly higher values than both *E. isabellinus* groups. *E. serotinus* had the longest CM3 (mainly allopatric *E. serotinus*), followed by the hybrids and *E. isabellinus* (sympatric and allopatric) (Table 1). Regarding C1-C1, hybrids showed the lowest values alongside the allopatric *E. isabellinus*, which was the only group from which hybrids did not significantly differ (Table S1). In the multivariate analysis, hybrids showed intermediated phenotypes in dentition, wing sizes (PC1 morphology) and the metacarpals and first phalanges proportion, as well as between the length of the first phalanges and the dentition (mainly CM3) (PC2 morphology).

**Table S1.** Statistical values of multiple comparisons of original morphometric variables according to the post-hoc Fisher’s LSD test. Green cells represent significant differences (P < 0.05).

**Fisher’s LSD**

|  |  | Eisa_all | Eisa_sy | Hybrid | Eser_sy | Eser_all |
| --- | --- | --- | --- | --- | --- | --- |
|  |  |  | FA | | | |
| Eisa_all |  |  | 0.826 | 0.451 | 0.008 | 0.006 |
| Eisa_sy | D3 MC | 0.026 |  | 0.535 | 0.013 | 0.010 |
| Hybrid |  | 0.524 | 0.670 |  | 0.253 | 0.317 |
| Eser_sy |  | 0.249 | 0.003 | 0.219 |  | 0.752 |
| Eser_all |  | 0.000 | 0.000 | 0.005 | 0.005 |  |
|  |  |  |  |  |  |  |
|  |  |  | D5 MC | | | |
| Eisa_all |  |  | 0.047 | 0.513 | 0.891 | 0.001 |
| Eisa_sy | D5 F1 | 0.085 |  | 0.771 | 0.066 | 0.000 |
| Hybrid |  | 0.011 | 0.080 |  | 0.483 | 0.029 |
| Eser_sy |  | 0.004 | 0.132 | 0.375 |  | 0.008 |
| Eser_all |  | 0.036 | 0.721 | 0.110 | 0.220 |  |
|  |  |  |  |  |  |  |
|  |  |  | CM3 | | | |
| Eisa_all |  |  | 0.254 | 0.000 | 0.000 | 0.000 |
| Eisa_sy | C1-C1 | 0.019 |  | 0.002 | 0.000 | 0.000 |
| Hybrid |  | 0.572 | 0.040 |  | 0.171 | 0.059 |
| Eser_sy |  | 0.003 | 0.301 | 0.009 |  | 0.563 |
| Eser_all |  | 0.004 | 0.593 | 0.017 | 0.554 |  |

**Multiple comparisons of colour variables**

All groups differed significantly from allopatric *E. serotinus* in the a* b* Grey-Brown scale (PC1 colour), being the only one in the grey space of the colour pallet. In terms of lightness (PC2 colour), allopatric *E. serotinus* was only not significantly different from individuals of the same species when in sympatry (they were equally dark although in sympatry they tended to be significantly browner). In sympatry both species also differed significantly, however, contrary to the morphometric variables, this difference was slightly higher than that found between species in allopatry. According to colour multivariate analyses, hybrids were browner than *E. serotinus* in allopatry and lighter than it both in sympatry and allopatry (Table S5). For the L* Lightness component (PC2 colour), hybrids demonstrated the lightest colour patterns (both in their dorsal and ventral coat) despite being not significantly different from both *E. isabellinus* groups (sympatric or allopatric) (Figure 3 and Table S5).

**Table S2**. Statistical values of multiple comparisons of dorsal and ventral original colour variables (L*, a* and b*). Green cells represent significant differences (P < 0.05).

**Fisher’s LSD**

|  |  | Eisa_all | Eisa_sy | Hybrid | Eser_sy | Eser_all |
| --- | --- | --- | --- | --- | --- | --- |
|  |  |  |  |  |  |  |
|  |  |  | L* dorsal | | | |
| Eisa_all |  |  | 0.423 | 0.318 | 0.004 | 0.015 |
| Eisa_sy |  |  |  | 0.618 | 0.000 | 0.006 |
| Hybrid |  |  |  |  | 0.045 | 0.120 |
| Eser_sy |  |  |  |  |  | 0.692 |
| Eser_all |  |  |  |  |  |  |
|  |  |  | b* dorsal | | | |
| Eisa_all |  |  | 0.474 | 0.240 | 0.043 | 0.152 |
| Eisa_sy | a* dorsal | 0.286 |  | 0.395 | 0.018 | 0.002 |
| Hybrid |  | 0.077 | 0.194 |  | 0.435 | 0.003 |
| Eser_sy |  | 0.010 | 0.005 | 0.501 |  | 0.000 |
| Eser_all |  | 0.045 | 0.000 | 0.000 | 0.000 |  |
|  |  |  |  |  |  |  |
|  |  |  | b* ventral | | | |
| Eisa_all |  |  | 0.747 | 0.311 | 0.298 | 0.007 |
| Eisa_sy | a* ventral | 0.938 |  | 0.283 | 0.196 | 0.000 |
| Hybrid |  | 0.581 | 0.486 |  | 0.885 | 0.000 |
| Eser_sy |  | 0.390 | 0.170 | 0.799 |  | 0.000 |
| Eser_all |  | 0.000 | 0.000 | 0.000 | 0.000 |  |

**Table S3.** Principal components extracted from the PCA analysis, with the respective eigenvalues, % variance explained, Cronbach‘s α and weight of each variable.

| Morphometric variables | Morphometry | | Colour | |
| --- | --- | --- | --- | --- |
|  | PC1  (Size) | PC2  (Shape) | PC1  (a*b* Grey-Brown) | PC2  (L* lightness) |
| FA | 0.973 | -0.071 | - | - |
| D3 MC | 1.020 | -0.159 | - | - |
| D3 F1 | 0.679 | 1.035 | - | - |
| D5 MC | 0.939 | -0.253 | - | - |
| D5 F1 | 0.759 | 0.757 | - | - |
| C1-C1 | 0.693 | -0.390 | - | - |
| CM3 | 0.656 | -1.030 | - | - |
| L* dorsal | - | - | -0.387 | 1.125 |
| a* dorsal | - | - | 1.084 | 0.139 |
| b* dorsal | - | - | 1.032 | 0.128 |
| L* ventral | - | - | -0.436 | 1.157 |
| a* ventral | - | - | 1.028 | 0.114 |
| b* ventral | - | - | 0.989 | 0.546 |
| *Eigenvalue* | 3.315 | 1.245 | 3.552 | 1.450 |
| Variance explained | 43.92% | 15.90% | 59.19% | 24.17% |
| Cronbach‘s α | 0.815 | 0.230 | 0.862 | 0.373 |

**Table S4.** Statistical values of multiple comparisons of means orders of morphometric PCA variables. Green cells represent significant differences (P < 0.05).

**Tukey’s LSD**

|  |  | Eisa_all | Eisa_sy | Hybrid | Eser_sy | Eser_all |
| --- | --- | --- | --- | --- | --- | --- |
|  |  |  | PC1 Size | | | |
| Eisa_all |  |  | 0.992 | 0.715 | 0.009 | 0.000 |
| Eisa_sy | PC2 Shape | 0.994 |  | 0.860 | 0.024 | 0.000 |
| Hybrid |  | 0.987 | 0.940 |  | 0.781 | 0.214 |
| Eser_sy |  | 0.267 | 0.124 | 0.889 |  | 0.732 |
| Eser_all |  | 0.002 | 0.000 | 0.328 | 0.727 |  |
|  |  |  |  |  |  |  |

**Table S5.** Statistical values of multiple comparisons of means orders of dorsal and ventral PCA colour variables. Green cells represent significant differences (P < 0.05).

**Fisher’s LSD**

|  |  | Eisa_all | Eisa_sy | Hybrid | Eser_sy | Eser_all |
| --- | --- | --- | --- | --- | --- | --- |
|  |  |  | PC1 a*b* Grey-Brown | | | |
| Eisa_all |  |  | 0.505 | 0.214 | 0.033 | 0.021 |
| Eisa_sy | PC2 L* Lightness | 0.883 |  | 0.315 | 0.008 | 0.000 |
| Hybrid |  | 0.578 | 0.532 |  | 0.409 | 0.000 |
| Eser_sy |  | 0.164 | 0.008 | 0.023 |  | 0.000 |
| Eser_all |  | 0.018 | 0.000 | 0.001 | 0.133 |  |
|  |  |  |  |  |  |  |

**Detail description of classification analyses**

**Species discrimination through classification statistics**

*1) Stepwise DFA with and without hybrids*

The morphometric stepwise DFA generated one discriminant function, retaining all variables as statistically significant (D3_F1 only marginally [F=3.643, *p=0.058*]). A single discriminant function was defined mainly by D3_MC and CM3. This function discriminated the two species (Λ=0.579, χ^2^_(7)_ = 53.773, p<0.001). The results of Classification Statistics showed that 49 out of 58 *E. isabellinus* individuals (84.5%) and 36 out of 46 *E. serotinus* individuals (78.3%) were classified correctly. The percentage of individuals classified correctly by stepwise DFA to morphometric variables in the presence of hybrids was 81.7%.

The morphometric stepwise discriminant analysis after hybrids removing generated also one discriminant function, with FA, D3_MC, D5_MC (marginally significant [F=3.450, *p=0.069*]) and CM3 as statistically significant variables. A single discriminant function was defined mainly by D3_MC and CM3. This function discriminated the two species (Λ=0.632, χ^2^_(4)_=21.073, p<0.001). The results of Classification Statistics showed that 30 out of 32 *E. isabellinus* individuals (96.0%) and 12 out of 18 *E. serotinus* individuals (66.7%) were classified correctly. The total percentage of individuals classified correctly by stepwise DFA of morphometric variables increased to 84.0% after hybrids removing.

The colour stepwise DFA generated also only one discriminant function, with L*_dorsal, a*_dorsal, b*_dorsal and L*_ventral as statistically significant variables. A single discriminant function was defined mainly by dorsal colour scales (a*_dorsal, L*_dorsal and b*_dorsal - in this decreasing order of influence). This function discriminated the two species (Λ=0.842, χ^2^_(4)_=10.115, *p=0.039*). The results of Classification Statistics showed that 36 out of 39 *E. isabellinus* individuals (92.3%), but only 9 out of 24 *E. serotinus* individuals (37.5%) were classified correctly. The percentage of individuals classified correctly by stepwise DFA to colour variables in the presence of hybrids was 71.4%.

After removing hybrids the colour stepwise discriminant analysis generated also one discriminant function, once again with L*_dorsal, a*_dorsal, b*_dorsal and L*_ventral as statistically significant variables. A single discriminant function was defined mainly by the variable L*_dorsal. This function discriminated the two species (Λ=0.762, χ^2^_(4)_=14.114, *p=0.007*). The results of Classification Statistics showed that 36 out of 39 *E. isabellinus* individuals (92.3%), but only 8 out of 17 *E. serotinus* individuals (47.1%) were classified correctly. The total percentage of individuals classified correctly by stepwise DFA of colour variables increased to 78.6% after hybrids removing.

*2) Support Vector Machine with and without hybrids*

SVM generated one discriminant function based on morphometric PC1 and PC2 as predictors’ variables that summarized all morphometric traits. Once composed of only two variables, a single classification function was defined similarly by PC1 and PC2 in the same direction. After simulations, the results of Classification Statistics showed that 45 of 57 individuals of *E. isabellinus* (78.9%) and 34 of 47 individuals from *E. serotinus* (72.3%) were classified correctly (Figure S2). The percentage of individuals classified correctly by SVM of morphometric variables in the presence of hybrids was 75.0%.

Without hybrids, SVM generated one discriminant function based on morphometric PC1 and PC2 as predictors’ variables that summarized all morphometric traits. Once composed of only two variables, a single classification function was defined especially by PC1. After simulations, the results of Classification Statistics showed that 26 of 31 individuals of *E. isabellinus* (83.9%) and 14 of 21 individuals from *E. serotinus* (66.7%) were classified correctly (Figure S2). The percentage of individuals classified correctly by SVM to summarized morphometric variables increased to 76.9% after removing hybrids.

SVM generated also one discriminant function based on colour PC1 and PC2 as predictors’ variables that summarized all colour variables. Composed of only two variables, a single classification function was defined mainly by PC1. After simulations, the results of Classification Statistics showed that 36 of 39 individuals of *E. isabellinus* (92.3%) and only 7 of 24 individuals from *E. serotinus* (29.2%) were classified correctly (Figure S2). The percentage of individuals classified correctly by SVM of colour variables in the presence of hybrids was 68.3%.

Without hybrids, the colour SVM generated one discriminant function based on PC1 and PC2 as predictors’ variables that summarized all colour variables. Once composed of only two variables, a single classification function is defined similarly by PC1 and PC 2 in opposite directions. The results of Classification Statistics showed that 37 of 48 individuals of *E. isabellinus* (77.1%) and only 2 of 8 individuals from *E. serotinus* (75.0%) were classified correctly (Figure S2). The percentage of individuals classified correctly by SVM of summarized colour variables increased to 76.8% after removing hybrids.

**Discriminant and classification functions**

1. *Stepwise DFA with hybrids*

*Morphometry*

Discriminant function (eigenvalue=0.726):

Species = 0.183 (FA) + 0.692 (D3_MC) – 0.468 (D3_F1) – 0.259 (D5_MC) – 0.359 (D5_F1) + 0.271 (C1_C1) + 0.837 (CM3).

Classification functions:

*E. isabellinus* = 16.760 (FA) + 6.148 (D3_MC) – 4.801 (D3_F1) – 3.999 (D5_MC) -6.861 (D5_F1) + 31.608 (C1_C1) + 25.371 (CM3) + 600.300

*E. serotinus* = 16.948 (FA) + 6.790 (D3_MC) – 5.576 (D3_F1) -4.214 (D5_MC) – 6.077 (D5_F1) + 30.035 (C1_C1) + 29.193 (CM3) + 643.292

*Colour*

Discriminant function (eigenvalue=0.187):

Species = 0.686 (L*_dorsal) – 0.825 (a*_dorsal) + 0.571 (b*_dorsal) + 0.111 (L*_ventral).

Classification functions:

*E. isabellinus* = 2.094 (L*_dorsal) + 13.092 (a*_dorsal) – 0.840 (b*_dorsal) + 2.243 (L*_ventral) – 131.706

*E. serotinus* = 1.979 (L*_dorsal) + 13.568 (a*_dorsal) – 0.922 (b*_dorsal) + 2.223 (L*_ventral) – 128.605

1. *Support Vector Machine with hybrids*

*Morphometry*

SVM function:

Species = - 4.59 (PC1_morphometry) – 4.08 (PC2_morphometry) + 0.02.

*Colour*

SVM function:

Species = - 4.39 (PC1_colour) – 0.28 (PC2_colour) - 0.42.

1. *Stepwise DFA without hybrids*

*Morphometry*

Discriminant function (eigenvalue=0.581):

Species = 0.026 (FA) + 0.501 (D3_MC) – 0.148 (D5_MC) + 0.815 (CM3).

Classification functions:

*E. isabellinus* = 21.383 (FA) + 6.178 (D3_MC) – 1.729 (D5_MC) + 34.384 (CM3) – 780.827

*E. serotinus* = 21.412 (FA) + 6.696 (D3_MC) – 1.850 (D5_MC) + 38.063 (CM3) – 829.241

*Colour*

Discriminant function (eigenvalue=0.312):

Species = 0.830 (L*_dorsal) – 0.395 (a*_dorsal) + 0.395 (b*_dorsal) + 0.182 (L*_ventral).

Classification functions:

*E. isabellinus* = 2.258 (L*_dorsal) + 12.308 (a*_dorsal) – 0.786 (b*_dorsal) + 2.193 (L*_ventral) – 131.525

*E. serotinus* = 2.054 (L*_dorsal) + 12.619 (a*_dorsal) – 0.851 (b*_dorsal) + 2.148 (L*_ventral) – 123.650

1. *Support Vector Machine without hybrids*

*Morphometry*

SVM function:

Species = -57.49 (PC1_morphometry) – + 25.91 (PC2_morphometry) – 2.97.

*Colour*

SVM function:

Species = -4.44 (PC1_colour) + 3.29 (PC2_colour) – 0.51.

|  | Morphometry | Colour |
| --- | --- | --- |
| With hybrids | 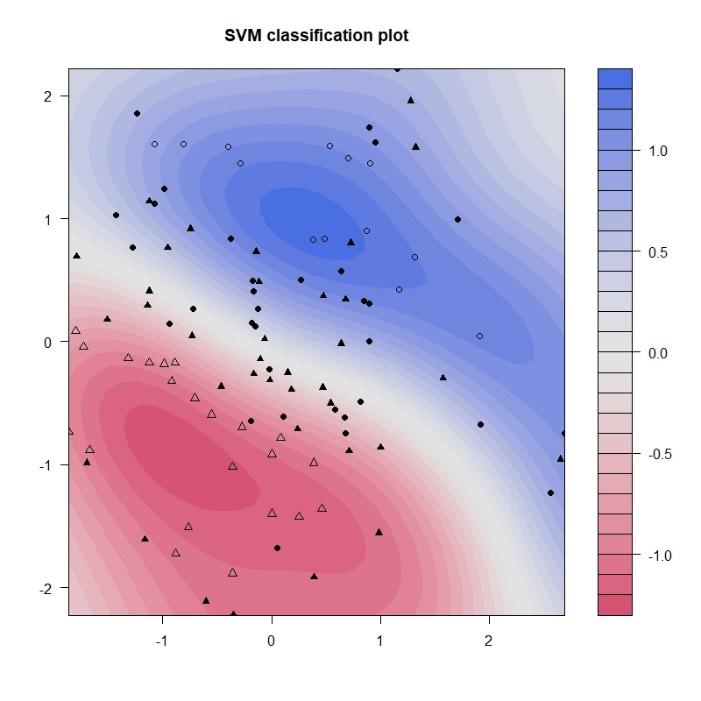 | 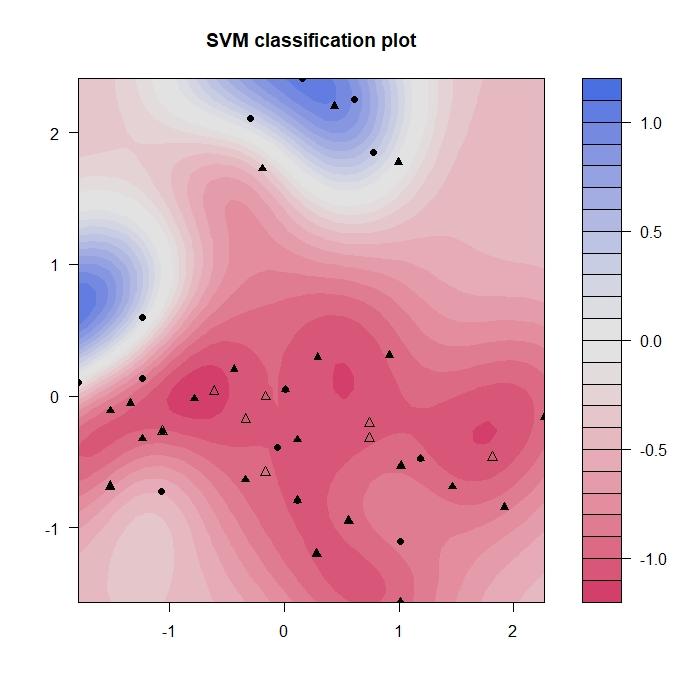 |
| Without hybrids | **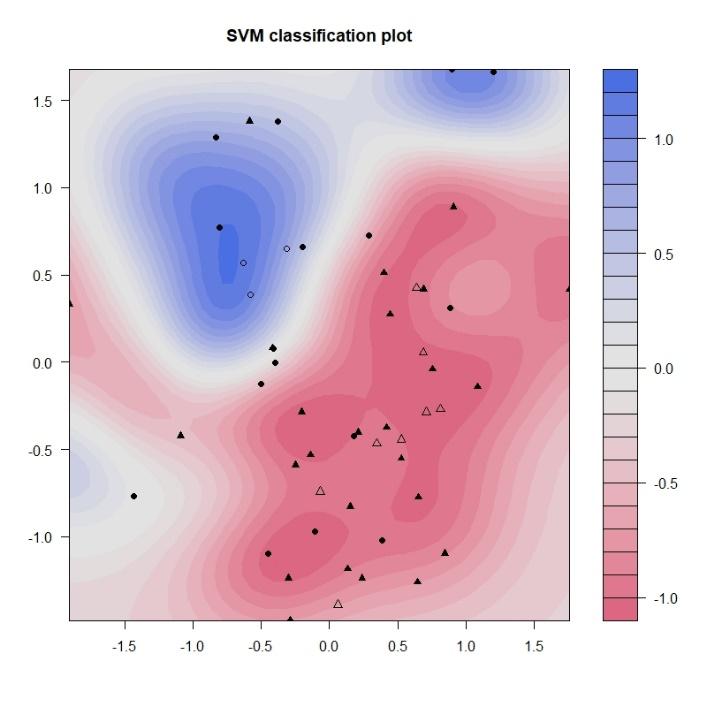** | **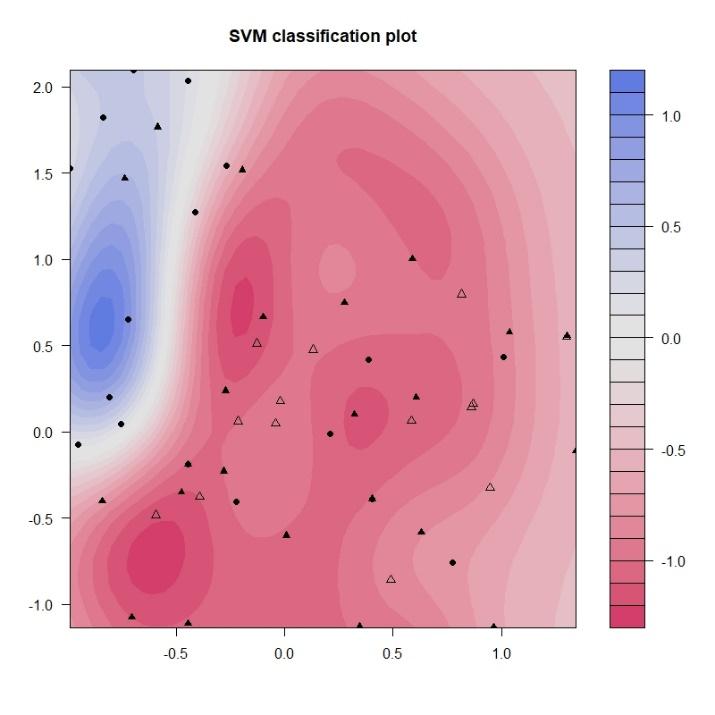** |

**Figure S3.** Support Vector Machine classification plots based on morphometric (on left) and colour traits (on right) for both parental species in sympatry, including hybrids on top and excluding them on the bottom. The density of probability of correct classification rate was based on Kernel statistics. Red spots regard *E. isabellinus* and the blue ones *E. serotinus*. Figure generated through “kernlab” and “e1071” R packages (v.3.6.2; <https://www.rstudio.com/>).
